# *Dirofilaria immitis* and *Angiostrongylus vasorum*: the contemporaneous detection in kennels

**DOI:** 10.1186/s12917-015-0619-y

**Published:** 2015-12-21

**Authors:** Luisa Del Prete, Maria Paola Maurelli, Saverio Pennacchio, Antonio Bosco, Vincenzo Musella, Lavinia Ciuca, Giuseppe Cringoli, Laura Rinaldi

**Affiliations:** Department of Veterinary Medicine and Animal Productions, University of Naples Federico II, CREMOPAR Campania Region, Via Della Veterinaria 1, 80137 Naples, Italy; Department of Health Sciences, University Magna Graecia of Catanzaro, Catanzaro, Italy; Jon Ionescu de la Brad”, University of Agricultural Sciences and Veterinary Medicine, Iasi, Romania

**Keywords:** *Dirofilaria immitis*, *Angiostrongylus vasorum*, Kennel, FLOTAC, DiroCHEK® ELISA

## Abstract

**Background:**

The cardiopulmonary nematodes *Dirofilaria immitis* and *Angiostrongylus vasorum* are increasingly reported in dogs and are responsible for two diseases with overlapping endemic areas, especially in Europe: dirofilariosis and angiostrongylosis. The reasons for their apparent emergence are unknown, but several factors (e.g. increased disease awareness, better diagnostic tools, climatic changes, seasonal population dynamics and movements of animals) may play a role in the recent rise in reports of infection in the various countries of Europe. The aim of this study was to investigate the seroprevalence of *D. immitis* (by DiroCHECK® ELISA) and the fecal presence of first stage larvae (L1) of *A. vasorum* (by FLOTAC) in dogs from 68 kennels of the Campania region (southern Italy). The fecal samples were collected from pooled samples using the box as epidemiological unit. To the authors’s knowledge, this is the first cross-sectional survey conducted at regional-scale in Italy and in Europe on the contemporaneous detection of *D. immitis* antigens and *A. vasorum* L1 in kennels.

**Results:**

Antigens of *D. immitis* were detected in 24/537 (4.4 %; 95 % Confidence Interval = 3.0–6.7) dogs in 6 out of the 68 kennels (8.8 %; 95 % CI = 3.6–18.9). The 24 positive samples for *D. immitis* antigen were tested also with AngioDetect® and only 1 sample was seropositive for *A. vasorum* with a prevalence of 4.2 %. *A. vasorum* L1 were detected in dogs from 9 out of the 68 kennels (13.2 %; 95 % CI = 21.8–44.9). Pooled fecal samples from 25 boxes out of the 1360 analyzed resulted positive to *A. vasorum* L1 (1.8 %; 95 % CI = 1.2–2.7).

**Conclusions:**

The present study indicates that cardiopulmonary nematodes are present in Campania region in symptomatic dogs as well as in asymptomatic ones. Therefore, regular parasitological surveillance, appropriate treatment strategies and high quality standard of hygiene are required to guarantee the health and welfare of kennel dogs.

## Background

The cardiopulmonary nematodes *Dirofilaria immitis* and *Angiostrongylus vasorum* are increasingly reported in dogs and are responsible for two diseases with overlapping endemic areas, especially in Europe: angiostrongylosis and dirofilariosis [1–3]. The reasons for their apparent emergence have been discussed in several recent studies; increased disease awareness, the availability of better diagnostic tools and climatic changes are considered the main causes of the recent spread of these parasites [2–7]. In particular, global warming represents a fundamental factor for the seasonality and the spread of *D. immitis* [7, 8] and could be involved also in the development of infectious stages in the intermediate hosts of *A. vasorum* [9, 10].

The adult stages of *D. immitis* and *A. vasorum* are both localized in the Arteria pulmonalis and the right heart of their definitive hosts [11]. *D. immitis* and *A. vasorum* cause different respiratory signs and severity may change in accordance to the parasite burden and host-related factors [12–14]. Age, immune response and presence of concomitant diseases can also determine fatal consequences in the infected animals [13, 14].

Current epidemiological data confirm the expanding trend of *D. immitis* in Italy, as observed in the rest of Europe [3, 15]. Today *D. immitis* is present not only in the hyperendemic area of the Po River Valley [3, 16], but from northern to southern regions of Italian peninsula as reviewed in Otranto et al. [17], including islands [18] where the infection is now endemic.

Similarly, *A. vasorum* is undoubtedly spreading from northern to southern Italy, especially in the central regions that offer ideal environmental and epidemiological conditions for the expansion of this parasite and the establishment of further new endemic foci [19, 20]. Knowledge of the epidemiology and clinical importance of *A. vasorum* has grown in the last 20 years and is correlated especially with increased urbanization of the red fox (*Vulpes vulpes*) which acts as a reservoir host representing a potential infection for dogs [21, 22].

In recent years, epidemiological surveys on *D. immitis* and *A. vasorum* prevalence have been conducted in many countries; however, no studies investigated the simultaneous detection of these cardiopulmonary nematodes in kenneled dogs. Therefore, the aim of this study was to investigate the distribution of *D. immitis* and *A. vasorum* in kenneled dogs of the Campania region of southern Italy. Noteworthy, to the authors’ knowledge, this is the first cross-sectional survey conducted at regional-scale in Italy and in Europe on the contemporaneous detection of *D. immitis* antigens and *A. vasorum* first stage larvae (L1) in kennels.

## Methods

### Study area and study kennels

The survey was conducted between June 2012 and August 2013 in the 68 public kennels located in the Campania region of southern Italy (www.anagrafecaninacampania.it). The region (Latitude = 39°59′15″–41°30′25″; Longitude = 13°45′25″–15°48′23″) which extends over an area of 13,590 km^2^ is mainly hilly and extends from 0 to 1890 m above sea level. The climate is Mediterranean with dry summers and rainy winters.

As in other Italian regions, the kennels of the Campania region are facilities for roaming and abandoned animals; dogs are sheltered and then given in adoption, if possible. Indeed, the Italian regulation for dog registration/identification (Law 281/1991) provides for setting up specific kennels to collect and re-home roaming and abandoned animals; for many disowned dogs, the kennel becomes a permanent shelter [23]. These kennels are subjected to veterinary public service control. Usually, few anthelmintic treatments per season are used in these kennels, with the number of treatments given per year ranging from 1 (15 %), 2 (75 %) to 3 (10 %) and using broad-spectrum antiparasitic drugs [24]. Routine prophylaxis for *D. immitis* is not performed in the region.

### *Blood sampling and analysis for* D. immitis *and* A. vasorum

In the 68 kennels, 537 blood samples (from 5 to 10 per each kennel) were collected. In each kennel we selected the dogs that were hosted for 2 years at least. The samples were transported to the laboratory and centrifuged at 3000 rpm for 10 min to obtain sera, then stored at -20 °C until testing for seroprevalence of *D.immitis* using DiroCHEK® ELISA (Synbiotics, San Diego, USA) according to the manufacturer’s instructions (sensitivity = 85–100 % and specificity = 100 %) [25].

After the analysis with DiroCHEK® ELISA, the dog sera resulted positive to *D. immitis* were also tested with the antigenic test AngioDetect® (IDEXX Laboratories, Westbrook, Maine, USA) (sensitivity = 84.6 %; specificity = 100 %) [26]. The blood samples were collected by the permission of the dog shelters. The animals used in the present study were sampled following approval by the animal ethics and welfare committee of the University of Naples Federico II (protocol number 0093412).

### *Fecal sampling and analysis for* A. vasorum

In each of the 68 studied kennels, the box (hosting a different number of dogs, i.e. min 1, max 4; mean = 2.4 dogs) was considered as the epidemiological unit of the study for practical reasons as reported in Rinaldi et al. [24]. In each kennel, 20 boxes were sampled by systematic sampling (total number of boxes = 1360). In each box, the fecal samples were collected directly from the ground (pooled samples), placed in a container and transported to the laboratory (within 5 h from sampling). The feces were fixed in 5 % formalin (dilution ratio 1:4). Copromicroscopic analyses were performed using the FLOTAC technique, having an analytic sensitivity of 2 larvae per gram (LPG) of feces. A zinc sulphate-based solution (specific gravity = 1.20) was used for diagnosis of *A. vasorum* [27].

## Results and discussion

Antigens of *D. immitis* were detected in dogs from 6 out of the 68 kennels (8.8 %; 95 % CI = 3.6–18.9). Specifically, 24/537 (4.4 %; 95 % CI = 3.0–6.7) dogs of 6 kennels were positive to *D. immitis* (Table 1). The 24 sera resulted positive to *D. immitis* were tested also with AngioDetect® in order to detect possible cross-reactions [11]. Only 1 sample (4.2 %; 95 % CI = 0.2–23.1) resulted positive for *A. vasorum* antigens. This dog did not show any clinical sign and *A. vasorum* L1 were detected in the corresponding box (see below).

**Table 1:** Prevalence of *Dirofilaria immitis* antigen at individual level in the positive kennels, *n* = 6

Kennel ID	No. of dogs examined	No. of dogs positive to *Dirofilaria immitis* antigen	Prevalence (%)	95 % confidence interval
1	20	5	25.0	9.6–49.4
2	93	5	5.3	2.0–12.7
3	46	3	6.5	1.7–18.9
4	94	6	4.2	2.6–13.9
5	94	4	4.2	1.4–11.2
6	9	1	11.0	0.6–49.3
Total	356	24		

*A. vasorum* (L1) were detected in dogs from 9 out of the 68 kennels (13.2 %; 95 % CI = 21.8–44.9; LPG min = 4; LPG max = 106; LPG mean = 25.1). Pooled fecal samples from 25 boxes out of the 1360 analyzed resulted positive to *A. vasorum* L1 (1.8 %; 95 % CI = 1.2–2.7). The mean number of *A. vasorum* positive boxes/kennel was 2.8 (min 1- max 7).

The strength of the present study was the contemporaneous detection of *D. immitis* antigens (8.8 %) and *A. vasorum* L1 (13.2 %) in kennels conducted by a cross-sectional survey at regional-scale.

However, also some limits emerged from our study. First, the lack of knowledge of the history of the kenneled dogs; we did not know if they were all natives of the Campania Region, even if autochthonous foci of canine *D. immitis* infection were recently reported in owned dogs from the same region [28]. Second, the impossibility to collect the blood from all the dogs examined by coprological techniques due to the aggressiveness of the kennel animals. Third, the using of pooled fecal samples; the percentage of positivity for *A. vasorum* found in this study (13.2 %) cannot directly be compared with prevalence of other study based on individual samples.

The main findings of the present survey confirm that polyparasitism is the rule in the studied kennels in southern Italy as already described by Rinaldi et al. [24] concerning other endoparasites. Different considerations emerged from this study related to: (i) epidemiology and (ii) diagnosis and control of cardiopulmonary nematodes infecting dogs.

*D. immitis* has spread in the last years in Europe due to climate change, the spreading of mosquito *Aedes albopictus* and the introduction of new vector *Aedes koreicus* [29]. These factors caused new autochthonous foci of heartworm disease that have recently been reported in previously non-endemic areas of southern Italy [8, 30]. While the distribution and prevalence of *D. immitis* infection has been widely studied in dogs from central and northern Italy, epidemiological data on the occurrence of *D. immitis* infection in southern Italy are scant and limited to sporadic case reports. The prevalence of *D. immitis* reported in this study was 8.8 %. This prevalence is higher than the previous study conducted in the Vesuvius area (0.6 %) of the same region confirming the spread of this parasite [28]. Similarly, also in Apulia and Calabria regions *D. immitis* was found in autochthonous dogs [17].

Different predictive studies, conducted using Geographical Information Systems (GIS) tools [15, 16], confirmed a correlation between the actual trend of *D. immitis* spread and the temperature increase into previously infection free areas as demonstrated also in a questionnaire survey by Genchi et al., [31] where an increasing number of positive cases to *D. immitis* was detected in non- endemic area (10 %) and in endemic- area (12 %) of Europe.

*A. vasorum* infection is considered endemic in certain areas of Europe, including regions of Denmark, Germany, Hungary, Finland, France, Ireland, Italy, the Netherlands, Poland, Slovakia, Spain, Sweden, Switzerland, Turkey and the United Kingdom. The country list is growing as the *A. vasorum* geographic distribution continues to evolve in several countries of Europe [13].

Our present study expands on that recently published by Rinaldi et al [32] which described a case of fatal disseminated *A. vasorum* infection in the same region. Also in the same region a prevalence of 33.3 % was found in red foxes at post-mortem examination [21] and the authors indicated the potential infection risk for dogs living in the same area. The most important and best-studied wildlife reservoir for *A. vasorum* infection is the red fox (*Vulpes vulpes*) [13, 22]. A possible explanation for increased transmission of infection between red fox and dog populations is an increasing density of foxes. It can be assumed that an increased density of foxes in an area populated with dogs is likely to increase the number of fox-dog interactions, and hence increase the opportunity for transmission of infection. Direct wild canid-dog interaction is not necessary for (and does not lead to) transmission of this parasite from one to the other because transmission occurs via ingestion of L3 (in gastropods or frogs, or possibly free in the environment) [13].

The positive percentage found in our present study (13.2 %), even if is not comparable with the prevalence of the other study because of the different epidemiological unit considered, is very high if compared other studies [33, 34]. The scenario of *A. vasorum* infection is alarming if we consider the clinical signs can be severe: respiratory dysfunctions, bleeding, neurological, ocular, cardiovascular and gastrointestinal symptomatology, skin lesions [12, 33]. Even if current data of this disease are referred to symptomatic dogs, *A. vasorum* infection may be also asymptomatic, especially in the early stages and so affected dogs can develop clinical signs after months to years from the infection [13].

Kennel dogs are at the highest risk of infection with helminths, for this reason anthelmintics or combinations of anthelmintics with a broad spectrum activity, as suggested in a previous study [24], are required to treat the polyparasitism currently encountered in this situations. Unfortunately, the anthelmintics used still now do not work as preventives of *D. immitis*. The reasons of the lack of a prophylaxis for *D. immitis* are: the unawareness of the severity of this parasite, the economic issue and the difficulties of managing and monitoring of the kenneled dogs.

## Conclusion

In conclusion, the present study indicates that the cardiopulmonary nematodes *D. immitis* and *A. vasorum* circulate in the kennels of the Campania region of southern Italy. Therefore, regular parasitological surveillance, appropriate diagnostic tools, treatment strategies and high quality standard of hygiene are required to guarantee the health and welfare of kennel dogs as recommended by the European Scientific Counsil for Companion Animals Parasites (www.esccap.org).

## Abbreviations

CI: confidence interval
L1: first stage larvae
LPG: Larvae per gram
